# Agent-based simulations of China inbound tourism network

**DOI:** 10.1038/s41598-019-48668-2

**Published:** 2019-08-23

**Authors:** Jinfeng Wu, Xingang Wang, Bing Pan

**Affiliations:** 1 School of Geography and Tourism, Shaanxi Normal University, Xi’an, 710062 China; 2 School of Physics and Information Technology, Shaanxi Normal University, Xi’an, 710062 China; 3 Department of Recreation, Park, and Tourism Management, School of Health and Human Development, Pennsylvania State University, University Park, Pennsylvania, 16802 USA

**Keywords:** Socioeconomic scenarios, Complex networks

## Abstract

Based on the results of a large-scale survey, we construct an agent-based network model for the China independent inbound tourism system and, by the approach of numerical simulation, investigate the responses of the tourist behaviors to perturbations in different scenarios, including the closure of a tourism city, the opening of a new port city in western China, and the increase of the tourism attractiveness of a specific city. Numerical results show that: (1) the closure of a single city in general will affect the tourist visitations of many other cities and, comparing to the non-port cities, the overall visitation volume of the system is more influenced by closing a port city; (2) the opening of a new port city in western China will attract more tourists to the western cities, but has a negligible impact on either the overall visitation volume or the imbalanced tourist distribution; and (3) the increase of the tourism attractiveness of a non-port (port) city normally increases (decreases) the overall visitation volume, yet there are exceptions due to the spillover effect. Furthermore, by increasing the tourism attractiveness of a few cities simultaneously, we investigate also the strategy of multiple-city-upgrade in tourism development. We find that the overall tourist volume is better improved by upgrading important non-port cities that are geographically distant from each other. The study reveals the rich dynamic inherent in complex tourism network, and the findings could be helpful to the development and management of China inbound tourism.

## Introduction

A well-known feature of tourism system is that cities inside the destination country form very complicated collaborative-competitive relationships^1,2^. On one hand, a tourism city acts not only as the destination, but also as the transition stops for the tourists to transfer to other cities. In this regard, the increase of the visitation of the destination will prompt the visitation of the transition city, i.e., their visitations are positively correlated and they form the collaborative relationship. On the other hand, due to practical concerns such as time limit and travel cost, a tourist can only visit a limited number of cities in the destination country. As a consequence of this, the increase of tourist visitation of a city could decrease the tourist visitation of another city. In this situation, the visitations of the cities are negatively correlated and they form the competitive relationship. The collaborative-competitive relationships between tourism cities render the dynamics of tourism system highly nonlinear, giving rise to many intriguing collective behaviors, e.g., a small, local perturbation added on a single city might trigger a large, global event over the whole system. The complicated relationships between tourism cities and the nonlinear feature of the system dynamics to perturbations pose unprecedented challenges for the development and management of tourism system, calling for the developments of new theoretical models and analyzing methods different from the traditional ones^3–6^.

There is an urgent need for China to develop the inbound tourism from the point of view of complex dynamical systems. As the 4th largest inbound-tourist destination in the world, the inbound tourism is an essential component of the China’s tourism industry^7^. However, comparing with the outbound tourism (China has been the largest resource of outbound tourists worldwide since 2009), the development of China inbound tourism is largely lagged behind^8^. Moreover, among the inbound tourists, a large fraction (about 81%) is contributed by Chinese tourists from Hong Kong, Macao, and Taiwan^8^. The dramatic imbalance between the inbound and outbound tourism markets makes the development of inbound tourism an economic imperative, which has been broadly interested and extensively studied by both the policy-makers and academic researchers in the past years^9–13^. Besides the overall tourist volume, another key challenge in developing the China inbound tourism is how to deal with the problem of imbalanced tourist distribution. According to the recent reports^14^, the tourism resources of China are concentrated in cites in the eastern part. These eastern cities, which are also densely populated and well developed in economy, attracted most of the inbound tourists (more than 80%)^15^. Despite stimulating efforts such as infrastructure investments and policy supports, the western cities are still lagging behind largely in terms of tourist visitation volume^16^. The imbalanced tourist distribution restricts seriously the performance of the whole inbound tourism, and how to eliminate the imbalanced distribution by attracting more tourists from the eastern to western cites has been one of the key issues for the development of a healthy China inbound tourism. To solve this problem, a common approach adopted currently in practice is to increase the tourism attractiveness of some western cities^16^, e.g., improving their service level and transportations. However, due to the complicated collaborative-competitive relationships between the tourism cities, the upgrading of a city may either prompt or deteriorate the whole market. To have a proper evaluation on the impacts of city upgrading, it is necessary to consider the networked tourism cities globally from the point of view of dynamical complex system and, more importantly, measure the impacts in a quantitative manner.

Network science provides a powerful tool for exploring the structure and performance of complex tourism systems^2,17^. Stimulated by the discovery of the small-world and scale-free features in many realistic networks^18,19^, in the past two decades great efforts have been paid to the exploration of the topological properties of various empirical networks, as well as their influences on the network functions^20^. As a typical example of complex social network, a variety of tourism network models have been constructed and studied in literature^21–25^. In the general model of tourism networks, the destinations are represented by nodes, and the interactions among destinations are represented by links. Depending on the specific problem that are interested, the network nodes and links may have different definitions. For instance, treating the tourism sectors (e.g., the tourism attractions, hotels, economic stakeholders, and service providers) within a destination city as nodes and the business relationship between them as links, in refs^17,22,23,26–29^ the authors have constructed the micro-tourism-network models. At the macroscopic level, a node can be defined as a country or a group of cities inside an area, and links can be defined as the economic ties among the counties or areas^21,25,30,31^. In previous studies of complex tourism networks, a common finding is that the networks possesses the general features of many realistic complex networks, e.g., the small-world property and heterogenous degree distribution^23,30^. It is worth noting that the existing studies focus mainly on the topological properties of the tourism networks, whereas their dynamical properties have been largely overlooked^17,21–31^. Specifically, it remains not clear how the patterns of the tourist flows are emerged from the random motions of the tourists, to what extend the tourism market is influenced if a destination city is closed during a tourism crisis, and how large the overall tourism performance will be increased if a tourist city is upgraded by the given amount of resources^26,31–38^. These dynamical properties, which are rooted in the collaborative-competitive relationships between the cities, are crucially important for the stable functioning of tourism network, as well as for the development and management of modern tourism.

In the present work, we construct an agent-based network model for the China inbound tourism system^13,15^, and, by the approach of numerical simulation, explore its dynamical responses to various external perturbations. In the constructed network model, each node stands for a specific tourism city in China, and two nodes are connected by a link if there is a direct transportation between the corresponding cities. We introduce a large number of agents into the network through only the port cities, and make the agents travel among the connected nodes in a random fashion. Based on the results of a large-scale survey, we obtain the key parameters characterizing the random movement of the agents, and then investigate numerically the responses of the tourist behaviors in different scenarios, including: (1) the removal of a city from the network (which simulates the closure of a city due to tourism crisis); (2) the opening of a new port city in western China (which models the adjustment of the tourism policy); and (3) the increase of city tourism attractiveness (which corresponds to city upgrading). Our main finding is that in a complex tourism network the performance of the tourism cities is strongly coupled with each other, and the change of a single city could affect the tourism of many other cities, resulting in a large variation in the overall tourism market. The study sheds lights on the dynamics of tourist flows in complex tourism system, and could be helpful to the development and management of China inbound tourism.

## Results

### Conceptual framework for the model of agent-based tourism network

The agent-based network model of China inbound tourism is constructed as follows. Firstly, we regard each destination city in China as a node. The network size, i.e., the total number of nodes in the network, is denoted as *n*. The cities are divided into two groups: the port and non-port cities. Here, port cities are defined as those cities where tourists are allowed to enter or leave China with certain probabilities, and non-port cities are defined as those cities where tourists can only pass through them. Correspondingly, the network nodes are classified into port and non-port nodes. Secondly, we attribute each city (node) a weight, *a*_*i*_, which characterizes the overall tourism attractiveness of the *i*th city (see Methods for the calculation of *a*_*i*_). Thirdly, a link is established between nodes *i* and *j* if the two cities are connected by either the direct flight or high-speed railway transportations. The link is weighted by the geographical distance, *d*_*ij*_, between cities *i* and *j*. We thus name ${\bf{D}}={\{{d}_{ij}\}}_{n\times n}$ the distance matrix, with $i,j=1,2,\ldots ,n$ the node indices. As ${d}_{ij}={d}_{ji}$, the network links are not directed. Finally, we input a large number of agents, *N*, into the network through the port nodes, and let the agents travel among the nodes in a random fashion. The agents are inputted and allowed to leave the network through the port nodes only. To be specific, denoting ${V}_{i}=\{l\}$ as the set of neighboring nodes connecting *i* in the network, the probability for an agent to be hopping from node *i* to *l* is defined as (the gravity model)^39,40^

1 $${p}_{il}=\frac{{a}_{l}^{\gamma }/{d}_{il}}{{\sum }_{l\in {V}_{i}}\,({a}_{l}^{\gamma }/{d}_{il})},$$

with *γ* a key parameter to be estimated from the surveys. We note that this model of agent-based complex tourism network is essentially different from the existing models in literature in that the tourists are moving over the network in a dynamic fashion, which is critical for mimicking the collaborative-competitive relationships among the tourism cities and the nonlinear responses of the network dynamics to external perturbations.

By numerical simulations, we monitor the movement of the agents on the network and keep records on their travel routes. When an agent arrives a port city, there is a predefined probability for it to leave the network. If the agent departs from this port city, the number of agents in the network will be deceased by one. The simulation is stoped once all agents leave the network. We then conduct analysis on the statistical characteristics of the travel routes (itineraries). We characterize each travel route by a chain of nodes, which are always started from and ended at the port nodes. For example, if an agent visited four cities in sequence, we denote its travel route by the chain: $A\to B\to C\to D$, with *A* the port city from where the agent enters the network, *D* the port city from where the agent leave the network, and *B* and *C* the transition cities the agent passes through. The length of the travel route is defined as $L=m+1$, with *m* the number of transition cities. For the example mentioned, we have $L=3$. The first statistical characteristic we are interested is the averaged route length, $\langle L\rangle ={\sum }_{l=1}^{N}\,{L}_{l}/N$. For the fixed number of agents, the larger is $\langle L\rangle$, the larger will be the overall visitation volume of the network. So the value of $\langle L\rangle$ reflects the overall performance of the system. The second statistical characteristic we are interested is the distribution of the tourist flows. Here, tourist flow is defined as the total amount of tourists passing through a specific link. For the link between nodes *i* and *j*, the tourist flow is denoted by *w*_*ij*_. It is noted that as *w*_*ij*_ includes both the routes from *i* to *j* and from *j* to *i*, we therefore have ${w}_{ij}={w}_{ji}$. We name ${\bf{W}}={\{{w}_{ij}\}}_{n\times n}$ the flow matrix. The third statistical characteristic we are interested is the visitation volumes of the cities, ${s}_{i}={\sum }_{j={V}_{i}}\,{w}_{ij}$, i.e., the total number of agents visiting node *i*. Different from $\langle L\rangle$, the matrices ${\{{w}_{ij}\}}_{n\times n}$ and ${\{{s}_{i}\}}_{n\times 1}$ capture the collective behaviors of the agents at the link and nodal levels, and give the detailed information of the tourist distribution over the network. It is worth noting that for the fixed number of agents, the three statistical characteristic are related with each other: $N\langle L\rangle ={\sum }_{i}\,{s}_{i}={\sum }_{i > j}\,2{w}_{ij}$.

### Empirical results obtained from questionnaire surveys

We concrete the conceptual model of tourism network by the results of a large-scale survey. In constructing the network model, we need to estimate from the surveyed data the following key parameters. First, we need to identify the set of cities contained in the network. Currently there are totally about 700 tourist cites in China, but only not all of them are included in our surveys. Second, as the behaviors of the tourists are very different at the port and non-port cities, we need to identify the set of port cities. Third, the fraction of tourists arriving a specific port city, as well as the probability for a tourist to depart from it. These information can only obtained from the questionnaires. With these concerns, in conducting the survey, we ask the surveyed tourists to provide the complete information of their itineraries, including the arrival, departure and transition cities (see Supplementary for an example of the completed questionnaires).

The questionnaire was translated into five different languages (English, Japanese, Korean, Russian, and French), and the survey was conducted from November 2010 to August 2011. The tourists were intercepted and surveyed at the major attractions of eight popular tourism cities in China, including Guangzhou, Shanghai, Hangzhou, Suzhou, Beijing, Chengdu, Guilin, and Xi’an. A total number of 3,000 questionnaires were distributed, with 2,687 returned on-site for a response rate of 89.6%. As we are focusing on only the behavior of independent foreign tourists, we keep only surveys from tourists arranging the trips by themselves, or by their relatives, employers or other individuals, rather than by tourism companies or travel agencies. For the same concern, we also remove the surveys from the grouped tourists, since tourists within a group do not make independent decisions. In addition, we remove the surveys collected from Chinese visitors (tourists from Hong Kong, Macao, and Taiwan), as they behave very differently from the foreign visitors^41^. After these operations, we have 1,451 effective questionnaires in total. Among them, 856 itineraries contain at least two cities, i.e., with the route length $L\ge 1$. These itineraries are the final samples used in our model construction and numerical analysis. An analysis of the final examples shows that the tourists came from 61 different countries and areas: 37.7% from Europe, 27.9% from Asian countries, 14.8% from Africa, 6.5% from North America, 8.2% from South America, and 4.9% from Oceania.

The itineraries contain totally $n=58$ tourism cities in China, including 4 port cites and 54 non-port cities. The four port cities are Beijing ($i=1$), Shanghai ($i=2$), Guangzhou ($i=3$), and Hong Kong ($i=4$), and their arrival fractions of tourists are ${p}_{1}^{a}=49.0 \%$, ${p}_{2}^{a}=26.4 \%$, ${p}_{3}^{a}=13.2 \%$ and ${p}_{4}^{a}=11.5 \%$, respectively. The probability for a tourist to depart from a port city is defined as the ratio between the number of departure tourists and the total tourist visitations of the port city. (In calculating the total tourist visitations of a port city, the number of arrival tourists has been excluded.) From the surveyed results, we have the following departure probabilities: ${p}_{1}^{d}=72.7 \%$ for Beijing, ${p}_{2}^{d}=57 \%$ for Shanghai, ${p}_{3}^{d}=48.6 \%$ for Guangzhou, and ${p}_{4}^{d}=51.1 \%$ for Hong Kong. In Table 1, we list all 58 tourism cities by the descending order of their tourist visitations (*s*_*i*_), together with their tourism attractiveness (*a*_*i*_).

**Table 1:** The tourism cities appeared in the questionnaires.

Index	City	Tourism attractiveness	Visitation volume	Index	City	Tourism attractiveness	Visitation volume
1	Beijing	224	970	30	Jiuzhaigou	36	14
2	Shanghai	141	864	31	Wenzhou	35	14
3	Guangzhou	76	529	32	Zhangjiajie	29	14
4	Hong Kong	62	492	33	Guiyang	27	12
5	Xi’an	66	467	34	Harbin	47	11
6	Guilin	96	446	35	Luoyang	50	10
7	Hangzhou	90	331	36	Shenyang	41	10
8	Chengdu	80	241	37	Xining	23	10
9	Suzhou	102	220	38	Yantai	52	10
10	Kunming	32	131	39	Changchun	16	8
11	Chongqing	180	110	40	Changsha	52	8
12	Nanjing	49	76	41	Zhuhai	6	7
13	Tianjin	82	71	42	Changzhou	34	6
14	Shenzhen	28	65	43	Fuzhou	30	6
15	Xiamen	38	53	44	Qinhuangdao	50	6
16	Dali	17	40	45	Dunhuang	18	4
17	Dalian	46	40	46	Hefei	54	4
18	Lijiang	28	38	47	Jingdezhen	20	4
19	Wuhan	58	36	48	Lhasa	16	4
20	Yiwu	47	36	49	Lanzhou	12	4
21	Datong	8	27	50	Nanchang	18	4
22	Huangshan	76	26	51	Nanning	36	4
23	Wuxi	75	26	52	Zhengzhou	32	4
24	Qingdao	65	24	53	Haikou	9	2
25	Urumqi	35	22	54	Xishuangbanna	29	4
26	Ningbo	86	18	55	Nantong	23	2
27	Shangri-La	20	16	56	Shantou	15	2
28	Sanya	38	15	57	Xuzhou	30	2
29	Jinan	38	14	58	Yichang	51	2

The cities are indexed by the descending order of their tourist visitation volumes, *s*_*i*_. Tourism attractiveness, *a*_*i*_, characterizes the overall attraction of a city. The four cities are Beijing, Shanghai, Guangzhou, and Hong Kong.

We next define the links connecting the nodes. A link is established between two nodes if there is at least one direct flight between the corresponding cities. The flight information and flight distance between the connected cities are obtained from the Civil Aviation Administration of China (CAAC)^42^. For cities that are not connected by direct flight, we check further the high-speed railway connections between them. The railway information is obtained from the Service Center of National Railway Administration of China^43^. By the time the surveys were conducted, there are totally 326 flight and high-speed railway connections among the investigated tourism cities. The connectivity of the cities is characterized by the distance matrix ${\bf{D}}={d}_{ij}$, with ${d}_{ij}={d}_{ji}$ the flight or railway distance between cities *i* and *j*.

**Figure 1:**
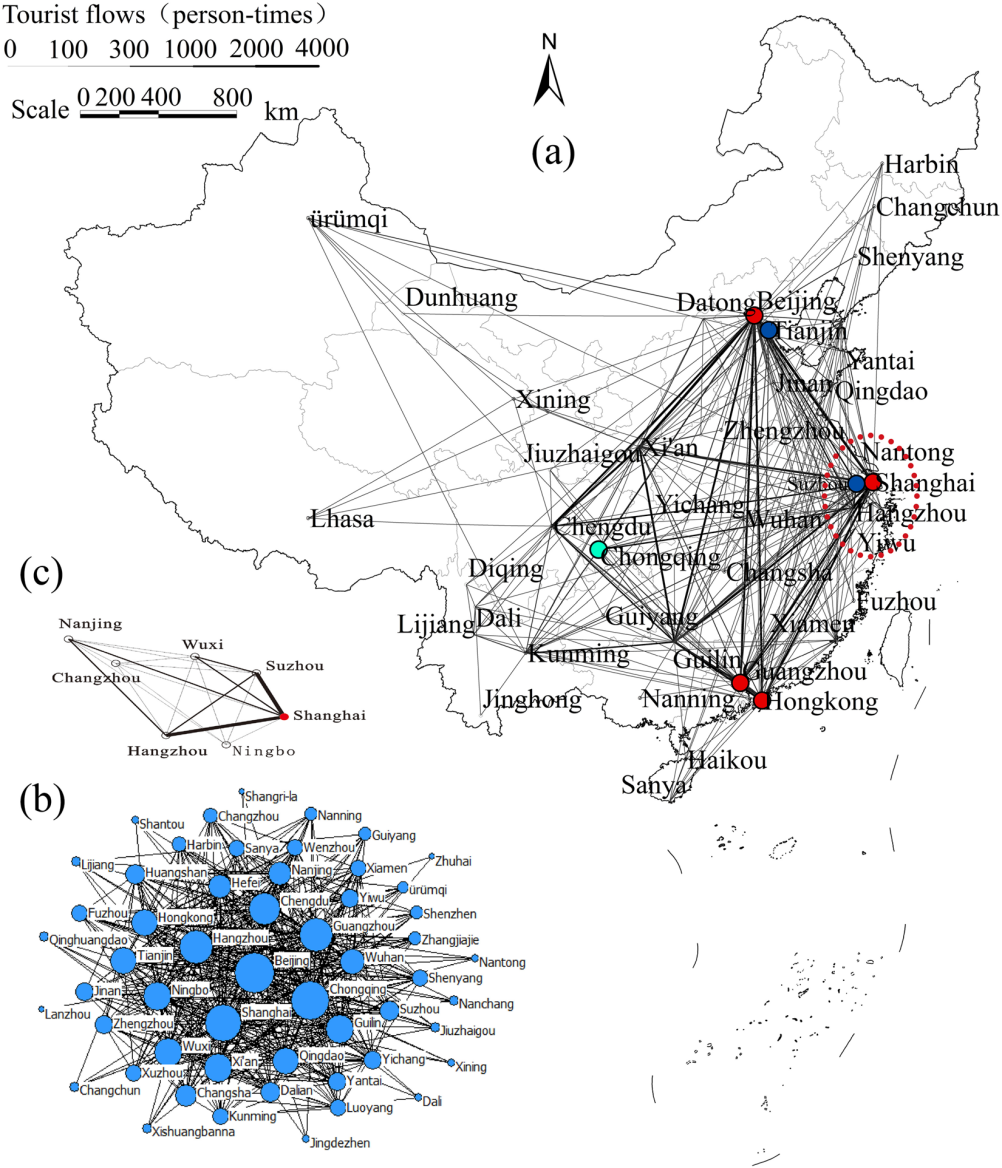
The China inbound tourism network constructed from the empirical and surveyed data. (**a**) The network structure, which consists of $n=58$ nodes (cities) and 2,135 links (flight and railway transportations). The thickness of the link is proportional to the tourist flow. The four port cities are colored in red. Chongqing is colored in green. Tianjin and Suzhou are colored in blue. Dashed ellipse denotes the Yangtze-River-Delta area. (**b**) The heterogeneous distribution of the city visitation volumes, {*s*_*i*_}. The size of node *i* is proportional to its visitation volume, *s*_*i*_. Port cities are colored in red. (**c**) The subnetwork formed by the cities inside the Yangtze-River-Delta area.

By the surveyed results, we plot in Fig. 1(a) the tourism network embedded in the geographical space. In Fig. 1(a), the volume of the tourist flow between cites *i* and *j*, *w*_*ij*_, is represented by the thickness of the associated link. We see that: (1) in consistent with the results in previous studies^23,30^, the tourism network possesses complex topological structure; (2) the distribution of the tourists is heterogeneous and imbalanced (most of the tourists are concentrated in the eastern part of China, especially in the well-developed regions such as the Yangtze-River-Delta and Pearl-River-Delta areas)^15^; (3) there are a few hub cities, e.g., the four port cities, which are densely connected to other cities in the network; (4) the tourist flows between the hub cities are clearly larger than those between the non-hub cities, forming the backbone of the network. The distribution of the tourist volumes, *s*_*i*_, are plotted in Fig. 1(b) by the core-periphery fashion, i.e., cities of larger visitation volumes are arranged at the core and those of small visitation volumes at the periphery. We see that, besides the four port cities (Beijing, Shanghai, Guangzhou, and Hong Kong), the visitation volumes of Hangzhou, Chengdu, Suzhou, Xi’an, and Guilin are also very large, signifying their importance to the system.

### Estimating the dynamical parameter of agent movement

Having fixed the properties of the network nodes (including the sets of port and non-port nodes, the arrival percentages (${p}_{1,2,3,4}^{a}$) and departure probabilities (${p}_{1,2,3,4}^{d}$) of the port cities, and the tourism attractiveness of each node (*a*_*i*_)] and links (i.e., the distance matrix ${\bf{D}}={\{{d}_{ij}\}}_{n\times n}$), we proceed to characterize the dynamical feature of the network, i.e., the moving fashion of the agents. Since both the node attractivenesses (${\{{a}_{i}\}}_{n\times 1}$) and link weights (${\{{d}_{ij}\}}_{n\times n}$) have been fixed according to the empirical data, the random movement of an agent, as described by Eq. (1), thus is dependent of the exponent *γ* only. In the present study, the parameter *γ* is trained by comparing the surveyed and numerical results, with the details the following. We scan *γ* over the range $(0,7)$ and, for each value of *γ*, compare the numerical results with the surveyed results for the following sample values: the route length (${\{{L}_{l}\}}_{N\times 1}$), the tourist flows (${\{{w}_{ij}\}}_{n\times n}$), and the visitation volumes (${\{{s}_{i}\}}_{n\times 1}$). For each of the sample values, the optimal value of *γ* is defined as the one that minimizes the difference between the numerical and surveyed results. We thus have three different optimal values for the exponent *γ*: ${\gamma }_{L}\approx 3.0$ for the route length, ${\gamma }_{s}\approx 2.3$ for the visitation volume, and ${\gamma }_{w}\approx 2.9$ for the tourist flow. The final value of *γ* used in Eq. (1) is chosen as the average of the three optimal values, which is $\gamma \approx 2.9$ (see Methods for more details about the estimation of *γ*).

### Numerical results based on agent-based simulations

With the constructed network model, we next investigate numerically the collective behaviors of the tourists in different scenarios, including: (1) to what extent will the overall tourist volume be affected when a single city is closed? (2) will the opening of a new port city in western China attract more tourists from the eastern cities? (3) how to improve efficiently the overall tourist volume by increasing the tourism attractiveness of one or a few cities? These questions capture the essential features of the collaborative-competitive relationships between cities in the tourism network, and are also of direct implications to the development and management of tourism^1–6,26,31–38^.

#### Removing a node

**Figure 2:**
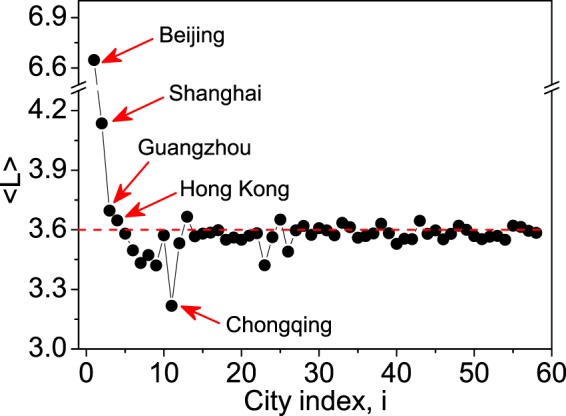
The impact of node closure on the average route length. By the results of numerical simulations, the variation of the average route length, $\langle L\rangle$, with respect to the index, *i*, of the removed city. Red dashed line denotes the value of $\langle L\rangle$ for the original network without node removal.

This scenario mimics the closure of a city during a tourism crisis, e.g., the outbreak of an epidemic disease. In numerical simulations, this is implemented by removing a city from the network, together with the links associated with it. If a port city is removed, its arrival agents will be distributed to other three port cities in proportion to their arrival volumes. We first investigate how the closure of a city will affect the overall tourist volume of the network, $S={\sum }^{}\,{s}_{i}$. As $S=N\langle L\rangle$ (with *N* the total number of agents and $\langle L\rangle$ the average route length), the value of *S* is determined solely by $\langle L\rangle$. Based on the numerical results, we plot in Fig. 2 the variation of $\langle L\rangle$ with respect to the index of the closed city, *i*. The results in Fig. 2 can be interpreted as follows. (1) The removing of a port city always increases the average travel length, therefore increasing the overall tourist volume. Comparing to Guangzhou ($i=3$) and Hong Kong ($i=4$), the average travel length is more significantly increased by removing Beijing ($i=1$) or Shanghai ($i=2$). (2) The removing of a non-port city may either increase or decrease the average travel length. However, comparing to the port cities, the overall tourist volume is only slight changed by removing a non-port city. (3) An exception in the non-port cities is Chongqing ($i=11$), where a sharp decrease of the average travel length is observed.

**Figure 3:**
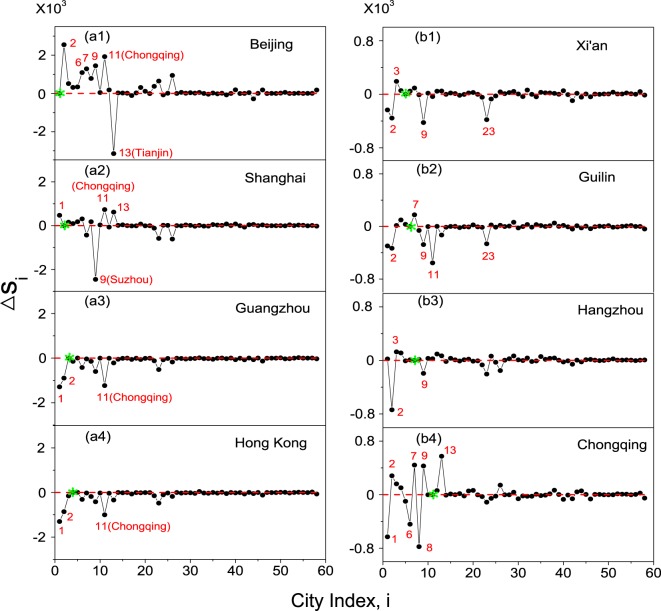
The influence of node removal on city tourist volumes. Left column: the results for the port cities. Right column: the results for four important non-port cities. ${\rm{\Delta }}{s}_{i}={s^{\prime} }_{i}-{s}_{i}$, with ${s^{\prime} }_{i}$ and *s*_*i*_ the visitation volumes of city *i* with and without the node removal. The removed city is marked by green asterisk in each panel. Please see Table 1 for the city indices.

To have more details on the influence of node closure on the tourist distribution, we plot in Fig. 3 the change of city visitation volume, ${\rm{\Delta }}{s}_{i}={s^{\prime} }_{i}-{s}_{i}$, when a specific city is removed. Here ${s^{\prime} }_{i}$ denotes the new visitation volume of the *i*th city. The results for removing port cities are plotted in Fig. 3(a1–a4). Figure 3(a1) shows that the removal of Beijing arouses a violent change of *s*_*i*_ to a few of other cities. By the descending order of Δ*s*, the top 5 cities that are benefited most from the removal of Beijing are Shanghai ($i=2$), Chongqing ($i=11$), Suzhou ($i=9$), Hangzhou ($i=7$), and Guilin ($i=6$). These five cities are located in different areas on the map [see Fig. 1(a)], signifying the global impact of Beijing on the whole tourism market. Interestingly, it is observed in Fig. 3(a1) that, different from other cities, the visitation volume of Tianjin ($i=13$) is dramatically decreased (${\rm{\Delta }}s\approx -\,3\times {10}^{3}$). The abnormal behavior of Tianjin can be attributed to the spillover effect of the tourists^44^. As the adjacent city of Beijing, Tianjin is normally chosen by the tourists as the transition stop. The two cities therefore form a strong collaborative relationship, which leads to the simultaneous increase or decrease of their visitation volumes. The results for removing Shanghai are plotted in Fig. 3(a2). We see that, similar to the results of Beijing, the removal of Shanghai increases the tourist visitations of a few of other cities too, although with the reduced scales. By the descending order of Δ*s*, the top 3 cities that are benefited most from the removal of Shanghai are Chongqing, Tianjin, and Beijing. Comparing with the results shown in Fig. 3(a1 and a2), we see that in the scenario of city closure, the visitation volumes of Beijing and Shanghai are negatively correlated. That is, the closure of one city will increase the visitation of the other city. Figure 3(a2) shows also that the visitation volume of Suzhou is mostly decreased when Shanghai is removed. Again, this phenomenon can be attributed to the spillover effect of the tourists, as Suzhou is in close proximity to Shanghai [see Fig. 1(a)]. It is worth noting that, different from other non-port cities, the visitation volume of Chongqing is significantly increased in both two cases, indicating the unique role of Chongqing in the network.

The results for the other two port cities, Guangzhou and Hong Kong, are presented in Fig. 3(a3 and a4), respectively. We see that, unlike Beijing and Shanghai, the removal of Guangzhou or Hong Kong arouses only moderate changes to the visitation volumes of a few other cities. Comparing the results presented in Fig. 3(a3 and a4), it is interesting to see that the responses of the other cities to the removal of Guangzhou are almost identical to that of Hong Kong. In both cases, the three mostly influenced cities are Beijing, Shanghai, and Chongqing. Particularly, the tourist visitations of Beijing and Shanghai are reduced by approximately the same amount. The results in Fig. 3(a3 and a4) suggest that Guangzhou and Hong Kong play a similar role in the network and, different from Beijing and Shanghai, their visitation volumes are positively correlated with each other. The similarity between Guangzhou and Hong Kong could be explained partially by their close proximity, and partially due to their remote distance from Shanghai and Beijing [see Fig. 1(a)]. In Fig. 3(a3 and a4), it is seen that the responses of Chongqing are distinctly large, justifying again the signifiant role of Chongqing in the network. Different from the cases of Beijing and Shanghai, here we see that the visitation volume of Chongqing is positively correlated with the visitation volumes of Guangzhou and Hong Kong, i.e., the decrease of the visitation volume of Guangzhou (or Hong Kong) will decrease the visitation volume of Chongqing.

We move on to evaluate the impact of removing a non-port city on the visitation volumes of other cities. Following the results in Table 1 and Fig. 2, we select Xi’an, Guilin, Hangzhou, and Chongqing as the targets. The first three cities are chosen for their importance in tourism, i.e., they have large visitation volumes (see Table 1), while Chongqing is chosen for its unique role in the network [as depicted in Figs 2 and 3(a1–a4)]. The results for removing Xi’an and Guilin are plotted in Fig. 3(b1 and b2), respectively. We see that the removal of Xi’an or Guilin has a modest impact on the visitation volumes of other cities. Figure 3(b3) shows the results for removing Hangzhou. We see that the visitation volumes of most of the cities are only slight changed, but the visitation volume of Shanghai is sharply decreased. The strong correlation between Shanghai and Hangzhou might be attributed to the spillover effect, as the two cities are adjacent on the map and the tourist flow between them is very large [see Fig. 1(a)]. The results for removing Chongqing are presented in Fig. 3(b4). Comparing to other three non-port cities, it is seen that the removal of Chongqing induces a violent changes to the visitation volumes of many other cities, especially for those important cities of large visitation volumes (of index $1 < i < 9$ in Table 1). The significant influence of Chongqing on the whole network, including the large decrease of $\langle L\rangle$ observed in Fig. 2 and the violent change of *s*_*i*_ in many cities observed in Fig. 3(b4), might be attributed to its unique geographical location (in the western part of China) and special topological structure (a hub node connecting all four port cities on the network).

In summary, our numerical studies on node removal show that: (1) the overall performance of the network is more influenced by removing a port city than a non-port city; (2) while the removal of a port city will increase the visitation volumes of many other cities, the visitation volume of its adjacent city however could be significantly decreased, due to the spillover effect (Tianjin to Beijing and Suzhou to Shanghai); (3) though as a non-port city, Chongqing is crucially important to the global performance of the network, i.e., the removal of it will induce a large reduction to the overall tourist volume (see Fig. 2), and its visitation volume is strongly coupled to the visitation volumes of many other cities (see Fig. 3).

#### Opening a new port city in western China

In solving the problem of imbalanced tourist distribution (i.e., most of the tourists are concentrated in the eastern part of China), a plausible approach would be upgrading one or a few of western cities, saying, for example, upgrading a non-port city to a port city. Before doing this, it is necessary to have a proper evaluation on the possible impact it will bring, e.g., the number of tourists that are attracted from the eastern cities and its impact on the overall visitation volume. As shown in Table 1, the two most influential cities in western China are Xi’an and Chengdu. These two cities thus are selected in our study as the candidates for upgrading. Also, for the unique role of Chongqing in the network, we also place this city on the list. In modeling, when a new port city is opened, we first need to assume two key parameters: the percentage of arrival tourists, ${p}_{new}^{a}$, and the departure probability of the tourists, ${p}_{new}^{d}$. Here, for the purpose of illustration, we set ${p}_{new}^{a}=10 \%$ and ${p}_{new}^{d}=40 \%$. [The arrival percentages of Beijing, Shanghai, Guangzhou and Hong Kong are adjusted to 35%, 25%, 15%, 15%, respectively, while their departure probabilities are kept as unchanged. For simplicity, we assume also that the opening of a new port city does not affect the total number of arrival tourists (see Discussions for the limitations of these settings)].

**Figure 4:**
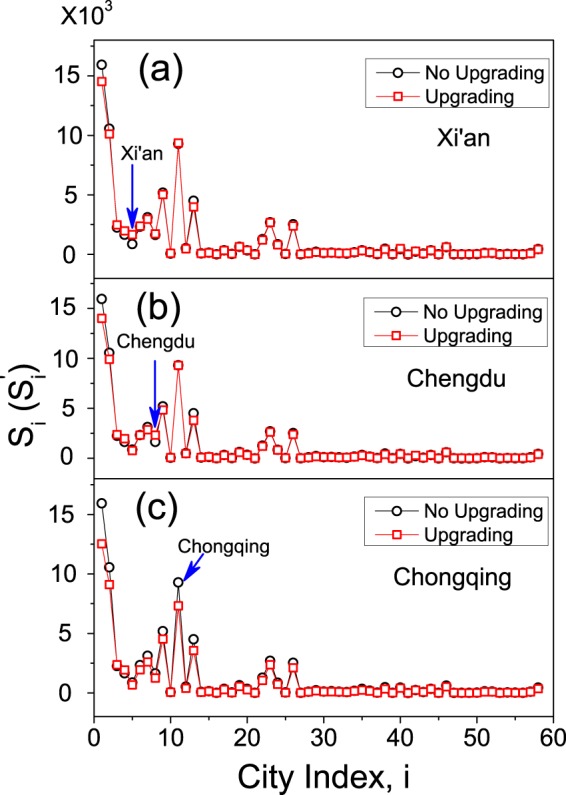
The impact of opening a new port city on the city visitation volumes. (**a**) Xi’an. (**b**) Chengdu. (**c**) Chongqing. Black circles: the original distribution (*s*_*i*_). Red squares: the distribution when a new port city is opened (${s^{\prime} }_{i}$).

We first check the change of the overall tourist volume, *S*, induced by opening a new port city. For the original network, the overall visitation volume is $S=68,302$. When Xi’an is upgraded as a port city, the overall visitation volume is increased to $S=65,956$. For Chengdu and Chongqing, the overall visitation volumes are changed to $S=65,104$ and $53,940$, respectively. Numerical simulations thus suggest that the opening of a new port city in western China has negligible impact on the overall visitation volume. Figure 4 shows the changes of the city visitation volumes, {*s*_*i*_}, when different cities are upgraded. We see that, comparing to the results without upgrading, the visitation volumes of the cities are almost unchanged in all cases. To quantify the overall impact of opening a new port city on the tourist distribution, we calculate the change of the geographic concentration index. The geographic concentration index is defined as^45^

2 $$G=100\times \sqrt{\mathop{\sum }\limits_{i=1}^{n}\,{({s}_{i}/\langle s\rangle )}^{2}},$$

with $n=58$ the number of nodes in the network and $\langle s\rangle =S/n$ the averaged city visitation volume. In general, the larger is *G*, the more heterogenous and imbalanced are the tourists distributed on the network. It is therefore desirable that by opening a new port city in western China the value of *G* could be significantly decreased, so that more tourists will be attracted from eastern to western cities. For the original network, we have $G=36.79$. By upgrading Xi’an, Chengdu and Chongqing as the port city, the value of *G* is changed to 33.01, 33.26 and 33.27, respectively. We see that by opening a new port city in western China, the value of *G* is only slightly decreased, indicating the infeasibility of this approach in balancing the tourist distribution.

In summary, the opening of a new port city in western China neither affect significantly the overall visitation volume, nor the tourist distribution. That is, the problem of imbalanced tourist distribution can not be solved effectively by opening a new port city in western China.

#### Increasing city tourism attractiveness

In tourism development, instead of a global upgrading (due to the limited resources), a common approach adopted in practice is increasing the tourism attractiveness of some specific cities each time, saying, for example, opening a new scenic area in a tourism city. While this approach in general could attract more tourists to the upgraded city, it may either increase or decrease the visitation volumes of other cities, due to the complex collaborative-competitive relationships between the networked cities. Furthermore, for the limited amount of upgrading resources, to which city should one invests such that the overall visitation volume of the system is maximally increased? Finally, when a large amount of upgrading resources are available, should one invests all the resources into a single city or, alternatively, distributes the resources among a few cities? We next address these questions by numerical simulations, based on the proposed model of agent-based tourism network.

**Figure 5:**
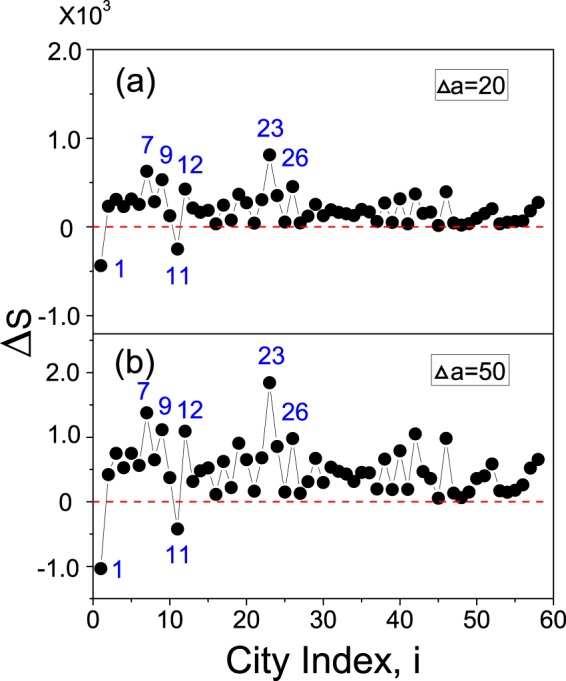
The impact of increasing the tourism attractiveness of a single city on the overall visitation volume. The increment of tourism attractiveness is fixed as ${\rm{\Delta }}a=20$ in (**a**) and ${\rm{\Delta }}a=50$ in (**b**). ${\rm{\Delta }}S=S^{\prime} -S$ denotes the change of the overall visitation volume.

We first evaluate the influence of upgrading a city on the overall visitation volume. Fixing the increment of the tourism attractiveness as ${\rm{\Delta }}a=20$ (which, according to the calculation of tourism attractiveness (see Methods), corresponds to adding 4 5A scenic areas in a city), we upgrade the cities individually and calculate the new overall visitation volume, *S*′. The variation of the overall visitation volume, ${\rm{\Delta }}S=S^{\prime} -S$, with respect to the city index is plotted in Fig. 5(a). We see that, except Beijing ($i=1$) and Chongqing ($i=11$), the upgrading of a city will always increase the overall visitation volume. By the descending order of Δ*S*, the top 5 cities are Wuxi ($i=23$), Hangzhou ($i=7$), Suzhou ($i=9$), Nanjing ($i=12$), and Ningbo ($i=26$). As shown in Fig. 1, these 5 cities are all located in the Yangtze-River-Delta area (the well-developed area in eastern China) and, more importantly, they form a strongly coupled motif which attracts a large fraction of the tourists (see Table 1). This observation seems to suggest that, to efficiently increase the overall visitation volume, the upgrading of a minor city belonging to a strongly coupled motif could achieve a better performance than upgrading a port or major city. The decreased overall visitation volume at Beijing may be attributed to its higher departure probability (${p}_{1}^{d}=72.7 \%$), which is clearly larger to that of other port cities (${p}_{2}^{d}=57 \%$ for Shanghai, ${p}_{3}^{d}=48.6 \%$ for Guangzhou, and ${p}_{4}^{d}=51.1 \%$ for Hong Kong). In fact, the impact of upgrading a port city on the overall visitation volume is double-edged. On one hand, the upgrading will attract more tourists to the upgraded city, which tends to increase the overall visitation volume. On the other hand, as the departure probability is keeping unchanged, there will be more tourists departing from the upgraded city, leading to the decrease of the overall visitation volume. If the latter plays the dominant role, the overall visitation volume will be decreased, e.g., the case of Beijing; otherwise, if the former is dominant, the overall visitation volume will be increased, e.g., cities such as Shanghai, Guangzhou, and Hong Kong. The sharp decrease of Δ*S* at Chongqing may attribute to its strong connections to the port cities. (Normally, when more tourists are attracted to Chongqing, the chance for them to travel to the port cities and then leave China will also be increased.) To check the generality of the results, we increase the increment of tourism attractiveness to ${\rm{\Delta }}a=50$ and plot in Fig. 5(b) the variation of Δ*S* with respect to the city index again. We see that the set of most influential cities are not changed with Δ*a*, and a sharp decrease of Δ*S* is also observed at Beijing and Chongqing.

**Figure 6:**
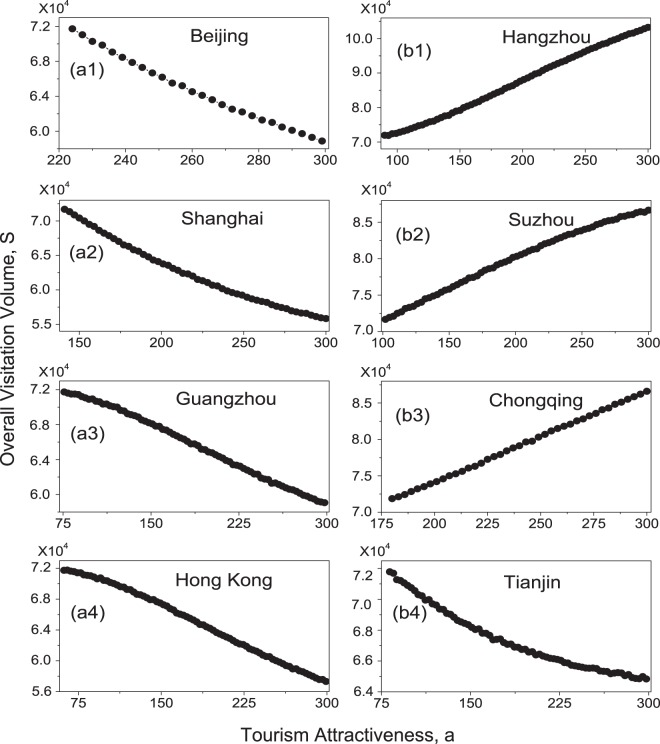
The variation of the overall visitation volume, *S*, with respect to tourism attractiveness, *a*, for the port cities (**a1**–**a4**) and several important non-port cities (**b1**–**b4**). (**a1**) Beijing. (**a2**) Shanghai. (**a3**) Guangzhou. (**a4**) Hong Kong. (**b1**) Hangzhou. (**b2**) Suzhou. (**b3**) Chongqing. (**b4**) Tianjin.

To explore further the influence of tourism attractiveness on overall visitation volume, we investigate the variation of *S* with respect to *a* over a wide range, saying increasing *a* from its current value to 300 for each city. The results for the port cities are plotted in Fig. 6(a1–a4). We see that, with the increase of *a*, the value of *S* is decreased monotonically for each case. This result is expectable, as by increasing the tourism attractiveness more tourists will be attracted to the port city, making the number of tourists departing from this city increased. Figure 6(b1–b4) show the variation of *S* with respect to *a* for some important non-port cities, including Hangzhou, Suzhou, Chongqing, and Tianjin. We seen that, except Tianjin, the value of *S* is monotonically increased with the increase of *a*, indicating the positive role of upgrading a non-port city in increasing the overall visitation volume. The abnormal behavior of Tianjin shown in Fig. 6(b4), again, can be attributed to the spillover effect of Beijing, as the visitation volumes of Tianjin and Beijing are positively correlated. This abnormal behavior, however, is absent when upgrading Suzhou, despite the spillover effect it receives from Shanghai. As depicted in Fig. 6(b2), with the increase of the tourist attractiveness of Suzhou, the overall visitation volume is monotonically increased. The behavior of Suzhou might be attributed to the strong couplings between Suzhou and other cities inside the Yangtze-River-Delta area, which reduce the fraction of tourists traveling to Shanghai.

When the tourism attractiveness of a non-port city is very large, it will be hard to increase the overall visitation volume by increasing *a*, as the impact of a single node on the whole network has its upper limit. This raises the question of upgrading efficiency, i.e., the increment of overall visitation volume per unit of increase of tourism attractiveness. Conventionally, the upgrading efficiency can be measured by the quantity ${S^{\prime} }_{slope}=|\partial S/\partial a|$. Generally, the smaller is the value of ${S^{\prime} }_{slope}$, the lower will be the upgrading efficiency. (For the port cities, the value of *S* is decreased by increasing *a*. In such a case, a smaller value of ${S^{\prime} }_{slope}$ represents a slower decrease of *S*). By scanning *a* over a wide range, i.e., from its current value to 1 × 10^3^, we calculate the variations of *S* with respect to *a* by upgrading different cities (see Supplementary), based on which the saturation points (defined as the point where ${S^{\prime} }_{slope}=0.1$) can be obtained. The saturation points of some major cities are listed in Table 2, together with the value of *S* at the saturation points. We see from Table 2 that the current tourism attractivenesses of the non-port cities are far below their saturation points, indicating their large potentials in tourism development.

**Table 2:** The development potentials of some major cities in China.

City	Current attractiveness	Saturation attractiveness	Saturation overall visitation volume
Xi’an	66	755	1.3 × 10^5^
Guilin	96	845	1.5 × 10^5^
Hangzhou	90	670	1.2 × 10^5^
Chengdu	80	910	2.1 × 10^5^
Suzhou	102	500	0.9 × 10^5^
Kunming	32	950	1.8 × 10^5^
Chongqing	180	900	1.1 × 10^5^

Saturation attractiveness is defined as the point where ${S^{\prime} }_{slope}=0.1$ in the variation of the overall visitation volume, *S*, with respect to the tourism attractiveness, *a*. See the text for more details.

**Figure 7:**
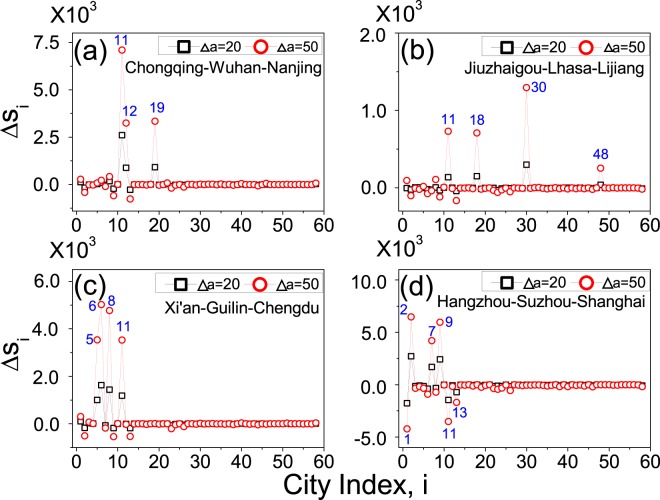
The performance of multiple-city-upgrade. The changes of city visitation volumes, ${\rm{\Delta }}{s}_{i}={s^{\prime} }_{i}-{s}_{i}$, by upgrading Chongqing-Wuhan-Nanjing (**a**), Jiuzhaigou-Lhasa-Lijiang (**b**), Xi’an-Guilin-Chengdu (**c**), and Hangzhou-Suzhouo-Shanghai (**d**). The tourism attractivenesses of the selected cities are increased by the same amount Δ*a*. Black squares: ${\rm{\Delta }}a=20$. Red circles: ${\rm{\Delta }}a=50$.

The saturation point in the variation of overall visitation volume reflects the limit of upgrading a single city in boosting the tourism market. When a large amount of upgrading resources are available, e.g., Δ*a* ~ 100, instead of upgrading a single city, a better performance might be achieved by distributing the resources among several cities, namely the strategy of multiple-city-upgrade. In applying this new strategy, a key question to be addressed is how to select the set of cities giving the best performance. To explore the performance of this new strategy in a qualitative manner, we fix the number of upgraded cities as 3, and check the performance of the following configurations in improving the overall visitation volume: Chongqing-Wuhan-Nanjing (across China from west to east), Jiuzhaigou-Lhasa-Lijiang (within a small area in western China), Xi’an-Guilin-Chengdu (within a broad area in western China), and Hangzhou-Suzhouo-Shanghai (within the Yangtze-River-Delta area in eastern China). For illustration purpose, we increase the attractiveness of the upgraded cities by the same amount. The changes of the city visitation volumes, ${\rm{\Delta }}{s}_{i}={s^{\prime} }_{i}-{s}_{i}$ with ${s^{\prime} }_{i}$ the updated city visitation volume, when upgrading Chongqing-Wuhan-Nanjing are plotted in Fig. 7(a). We see that except the three upgraded cities (*i* = 11, 12, 19), the visitation volumes of the other cities are almost unchanged. As the visitation volumes of the three upgraded cities are clearly increased, the overall visitation volume is still increased by a noticeable amount, as depicted in Table 3. An interesting finding here is that if the same amount of resources are concentrated in a single city, e.g., Nanjing (which generates the large increment in *S* among the three cities in terms of single-city-upgrade, as can be seen from Fig. 5), the increment of the overall visitation volume is much smaller to that of multiple-city-upgrade (see Table 3).

**Table 3:** The performance of multiple-city-upgrade.

City-configuration	*S*_*original*_	*S*_Δ *a*=20_	*S*_Δ *a*=50_
Chongqing-Wuhan-Nanjing	68,302	75,819	84,313
Jiuzhaigou-Lhasa-Lijiang	68,302	72,211	74,163
Xi’an-Guilin-Chengdu	68,302	76,451	86,871
Hangzhou-Suzhou-Shanghai	68,302	72,097	72,457

Each configuration contains three cities, and the selected cities are upgraded by the same amount of tourism attractiveness, Δ*a*. *S*_*original*_, *S*_Δ*a*=20_ and *S*_Δ*a*=50_ denote, respectively, the overall visitation volume without upgrading, with Δ*a* = 20 and Δ*a* = 50.

The results for Jiuzhaigou-Lhasa-Lijiang configuration are plotted in Fig. 7(b). We see that, besides the upgraded cities (*i* = 18, 30, 48), the visitation volume of Chongqing (which is not directly upgraded in this case) is also increased significantly. The results for Xi’an-Guilin-Chengdu configuration are plotted in Fig. 7(c). We see that, like the Jiuzhaigou-Lhasa-Lijiang configuration, besides the directly upgraded cities (*i* = 5, 6, 8), the visitation volume of Chongqing is also increased significantly. However, comparing with the results shown in Fig. 7(b), we see that the visitation volumes of the upgraded cities are increased more significantly in Fig. 7(c). As a result of this, the overall visitation volume is significantly increased by the Jiuzhaigou-Lhasa-Lijiang configuration (see Table 3). Figure 7(d) shows the results for the Hangzhou-Suzhouo-Shanghai configuration. Different from the other three configurations, we see that in this case the visitation volumes of Beijing ($i=1$), Chongqing and Tianjin ($i=13$) are clearly decreased. As a matter of fact, among the four tested configurations, the Hangzhou-Suzhouo-Shanghai configuration generates the smallest increment in the overall visitation volume, as shown in Table 3.

The results in Fig. 7 and Table 3 indicate that in applying the multiple-city-upgrade strategy, the target cities should be chosen not only by their local properties (e.g., the tourism attractiveness and visitation volume of an individual city), but also by their global features (e.g., the geographical location and node degree of each city). To be specific, numerical simulations suggest that the more important are the individual cities (with larger attractiveness and visitation volume) and the boarder are the cities distributed on the map, the larger will be the increase of the overall visitation volume when multiple-city-update is applied. The Jiuzhaigou-Lhasa-Lijiang and Hangzhou-Suzhou-Shanghai configurations take advantages from the former; the Chongqing-Wuhan-Nanjing configuration takes advantage from the latter; while the Xi’an-Guilin-Chengdu configuration takes advantages of both factors.

Summarizing the influence of increasing city tourism attractiveness on tourism, it is found that: (1) the increase of tourism attractiveness of a non-port (port) city in general will increase (decrease) the overall visitation volume; (2) the behavior of Tianjin is different from other non-port cities, due to its strong connection with Beijing; (3) each non-port city has a saturation point in the tourism attractiveness over which the overall visitation volume is hardly increased; (4) when a large amount of upgrading resources are available, the upgrading of several cities could increase the overall visitation volume more efficiently than upgrading just a single city.

## Discussions

In the present study, based on the results of large-scale survey, we have constructed an agent-based network model for the independent inbound tourism system of China. With this model, we have investigated numerically the responses of the tourist flows in some scenarios of practical significance, including the closure of a city, the opening of a new port city in western China, and the upgrading of city tourism attractiveness. Our main findings are:

1. The closure of a single city in general will affect the visitation volumes of many other cities in the network, due to the collaborative-competitive relationships between the networked cities. Comparing to a non-port city, the overall visitation volume is more influenced by the closure of a port city. Whereas the closure of a port city could increase the overall visitation volume, there are cities in the network whose visitation volumes are significantly decreased due to the closure, such as Tianjin (when Beijing is closed) and Suzhou (when Shanghai is closed). The abnormal responses of Tianjin and Suzhou are attributed to the spillover effect of the port cities. Among the non-port cities, the behavior of Chongqing is very special in that it is strongly coupled to many important cities in the network, and the closure of Chongqing will generate a significant decrease in the overall visitation volume.
2. The opening of a new port city in western China will attract more tourists to the western cities, but can not solve fundamentally the problem of imbalanced tourist distribution. As a matter of fact, the opening of a port city in western China will decrease slightly the overall visitation volume. To balance the tourist distribution, a more efficient approach would be increasing the attractiveness of several important cities in western China together. Numerical simulations suggest that with the same amount of upgrading resources, the latter is more efficient in attracting tourists to western China and, in the meantime, is able to increase the overall visitation volume.
3. The increase of tourism attractiveness of a port (non-port) city in general will decrease (increase) the overall visitation volume. Cities inside the Yangtze-River-Delta area form a strong motif, and behave coherently to external perturbations. More importantly, while individually each city in this motif is not important (of larger index in Table 1), the strong couplings between them render each city with great potential in tourism development, i.e., the increase of the attractiveness of any city in the motif could generate a large increment to the overall visitation volume. An interesting finding is that the upgrade of multiple cities could be more efficient in prompting the market than upgrading a single city. In choosing the cities to be upgraded, both their individual importance (i.e., tourism attractiveness and visitation volume) and network features (i.e., geographical location and node degree) should be taken into account.

### Implications

The above findings could be helpful to the development and management of inbound tourism), including:

1. Shedding new lights on the dynamical behavior of tourism system. Our numerical studies demonstrate that, due to the collaborative-competitive relationship among the cities, the tourism markets of the tourism cities are not isolated from each other, but are coupled tightly in a dynamic fashion. In particular, it is shown that in some scenarios, e.g., the removal of an important city, the change made on a single city could trigger a large-scale response involving many other cities in the network. Numerical evidences also show that, besides the intrinsic city properties (e.g., the tourism attractiveness), the importance of a city to the whole network is also valued by its structural properties, including the geographical location, node degree, and the set of adjacent cities. These features, which are absent in the model of static and regular tourism networks, manifest the necessary of treating tourism system as a dynamical, complex network.
2. Providing a quantitative evaluation on the impacts of external perturbations on tourism performance. Whereas it is well noticed that in tourism system a local perturbation could induce a global response, a quantitative evaluation on the degree and scale of such a response is still missing. With the agent-based network model, we are able to quantify not only the degree of the global response (e.g., the variation of the overall visitation volume), but also the detailed changes at the city level (e.g., the changes of the city visitation volumes). In addition, by the agent-based network model, we are also able to quantify the degree of correlations between the cities, based on which the spillover effect of Beijing on Tianjin (and also Shanghai on Suzhou), the synchronous behavior of Guangzhou and Hong Kong, and the crucial role of Chongqing have been revealed.
3. Proposing a new strategy for tourism development. Our numerical results show that neither the opening of a new port city nor the increase of the tourism attractiveness of an individual city in western China will change the imbalanced tourist distribution. Our results of agent-based simulations suggest that, to attract more tourists to western China, an efficient approach would be upgrading simultaneously several cities that are deliberately selected according to several factors, including the city importance, connectivity, and geographical location. This finding is of practical significance as it points out the fact that in developing the tourism industry, the tourism cities should be upgraded from the global point of view, instead of their individual interest.

### Limitations and future research

The agent-based network model is constructed based on several key assumptions, which limit the accuracy and applicability of the results we have obtained. The following lists a few limitations of the proposed model, as well as questions that should be addressed by further studies.

1. The number of inbound tourists should not be fixed as constant. For simplicity, we have assumed in the model that when a city is closed or upgraded, the total number of agents inputted into the system is keeping unchanged. In a realistic situation, e.g., the closure of a city due to tourism crisis, the total number of tourists arriving China in general will be decreased. The similar concern exists also when increasing the tourism attractiveness of a city, which generally attract more inbound tourists. For instance, during the period of the 2018 Summer Olympic Games^34^, a large amount of foreign tourists were poured into Beijing. Besides, the number of inbound tourists is also varying with seasons and is affected by the performance of the global economy. Therefore, to make the model more realistic, the number of agents in simulations should be time- and perturbation-dependent.
2. More factors should be included in calculating the city tourism attractiveness, and more details about the tourist travel behavior should be considered. In our model, the attractiveness of a city is determined by only its 4A and 5A scenic areas. In the realistic situation, the city attractiveness is a global tourism indicator incorporating many elements, including economy level, hotels, services, and, of course, tourism attractions. In terms of the tourist travel behavior, we have assumed that the tourists are traveling independently in the network with the probability defined by Eq. (1). Although the travel routes are made by the tourists independently, there is still the possibility that the tourists interact with each other in an indirect fashion. For instance, a tourist may adjust his/her travel route after reading the travel logs posted by other tourists on websites such as Facebook and Twitter. Also, for the sake of simplicity, we have not considered in the model the tourist capacity of each city, which in realistic situation is also an important issue influencing the tourist behavior. When tourist capacity is considered, a city will not adopt new tourist once its visitation volume reaches the capacity. Finally, in determining the exponent *γ* in Eq. (1), we have defined it as the average of the optimal values obtained from three different distributions (i.e., the travel lengths, city visitation volumes, and tourist flows). While this definition is meaningful from the point of view of theoretical study, the realistic situation is far more complicated than this. Future studies taking into account these issues will be important and necessary.
3. The parameters of the model, either trained from the surveyed results or assumed based on experience, are just for the purpose of demonstration, which should be improved by further empirical studies. In estimating the key parameter *γ* in Eq. (1), we have adopted simply the average of three optimal values, namely *γ*_*L*_, *γ*_*w*_ and *γ*_*s*_. While these optimal values reflect the collective behavior of the tourists from different aspects, they are essentially related with each other. However, due to the limited surveys and small network size, we are not able to derive such a relation explicitly. Furthermore, besides the analyzed distributions, there could be also other statistical quantities that are interested in practical applications. In such a case, the value of *γ* should be redefined according to the specific question that is interested. The similar concerns arises also when opening a new port city in western China, in which we have set artificially the parameters, including the fraction of arrival tourists, the arrival percentage and departure probability. Certainly, by changing these parameters, the numerical results, e.g., the variations of the geographic concentration index and the overall visitation volume, will be modified. Finally, for the sake of simplicity, we have weighted the network links by the geographical distances. Other options such as weighting the links by the minimum traveling times will modified the results quantitatively, but we speculate that the main phenomena we have observed will be kept unchanged. It is our hope that by employing more sophisticated analyzing methods and additional empirical data, these parameters could be accurately defined. Nonetheless, our model of agent-based tourism network provides a solid step for the quantitative analysis of the dynamical behavior of complex tourism system.
4. Another question that has not been addressed in the present work is the impact of adding a new link. Recent studies in network science have shown that the introduction a shortcut link may change largely the network dynamics, which makes it interesting to identify the crucial link to be established between cities such that the system performance can be significantly improved. In practice, this scenario may correspond to the situation of opening a new flight or train between two cities. While the importance of a link in principle can be evaluated by its centrality, the current studies on link centrality are mainly focused on networks of homogeneous nodes. In our model of tourism network, the nodes are characterized not only but their attractiveness, but are also classified as port and non-port cities. These features render the current knowledge of link centrality can not be applied directly to tourism network. To find the crucial link, the only possibility seems to scan all the candidates (~*n*^2^), i.e., by the method of brute-force search. This method, however, requires a significant amount of computing resources, especially for the large-size networks. We wish this question could be addressed in our further studies.

## Methods

### Calculating the city tourism attractiveness

In our study, the tourism attractiveness of a city is calculated from its tourism attractions, as follows. The tourism scenic areas in China are ranked from 1A to 5A by the descending order of aesthetic quality. Among them, the 5A and 4A scenic areas are evaluated based on a national standard, namely the Quality Ranking and Evaluation of Tourism Scenic Areas, which are well advertised and propagated by the Ministry of Culture and Tourism of China. The scenic areas with ranks from 1A to 3A, however, are evaluated by the provincial tourism bureaus on behalf of the national committee, which are mainly targeted for domestic tourists. As we are interested in the behaviors of the inbound tourists, we therefore consider only tourism attractions of ranks 4A and 5A in calculating the tourism attractiveness. By the time the surveys were conducted, there are totally 117 5A scenic areas and 1,849 4A scenic areas in China. For the set of cities (58 in total) consisting of our tourism network model, there are totally 105 5A scenic areas and 783 4A scenic areas.

The weights of the scenic areas are obtained by the Delphi method. Specifically, we surveyed all faculty members (6 full, 6 associate, and 6 assistant professors) in the college of the first author, and conducted four rounds of surveys in total. We adopted the integers for all rounds. In the final results, one 5A scenic area was assigned a value of 5 and one 4A scenic area was assigned a value of 3. The full list of the tourism attractiveness of the investigated cities is given in Table 1.

### Estimating the parameter *γ* based on the surveyed results

**Figure 8:**
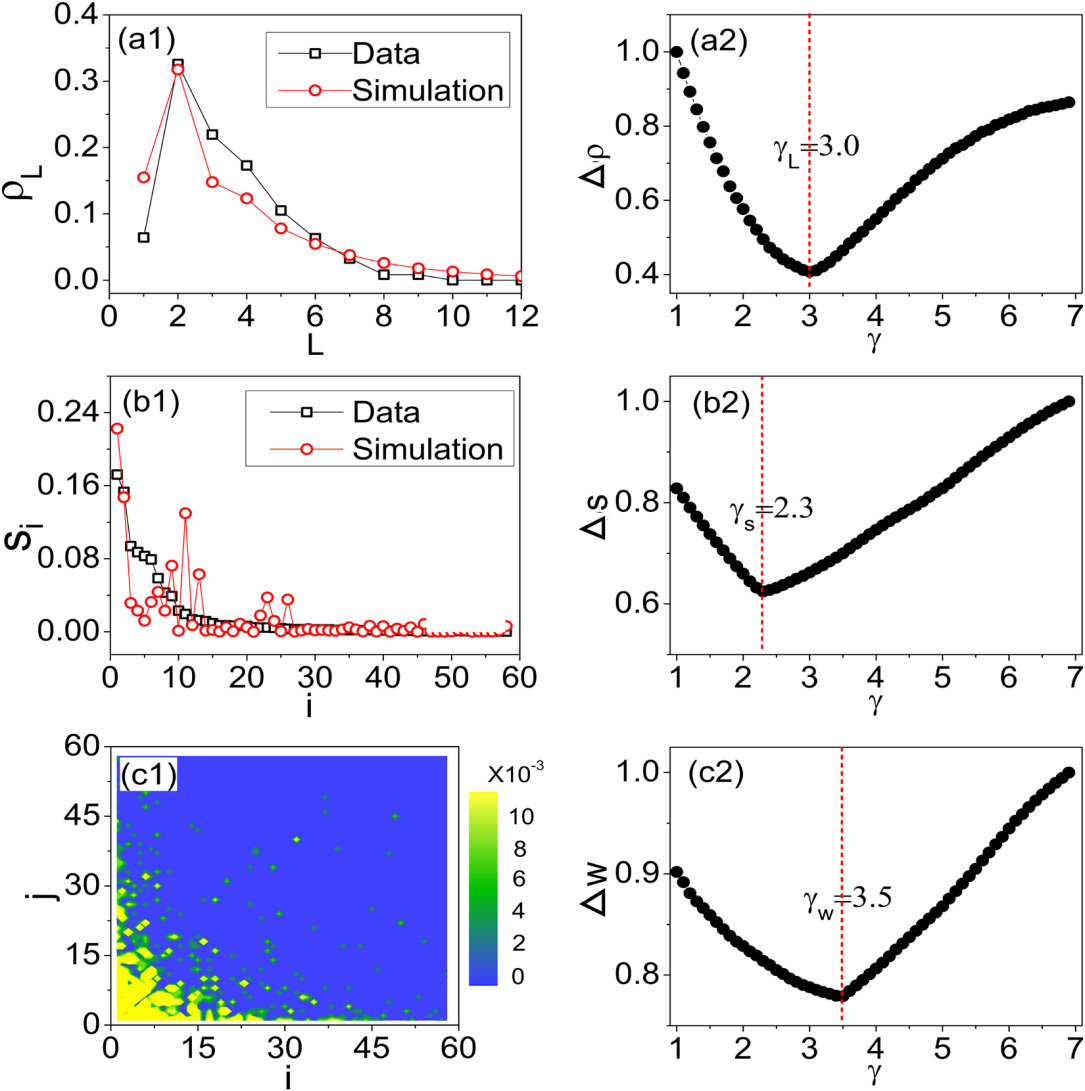
The estimation of parameter *γ*. (**a1**) The probability distribution of the travel length. Black squares: surveyed results. Red circles: numerical results obtained at *γ*_*L*_. (**a2**) The variation of the normalized difference of travel length, ${\rm{\Delta }}\rho$, with respect to *γ*. ${\rm{\Delta }}\rho$ is minimized at ${\gamma }_{L}\approx 3.0$. (**b1**) The distribution of city visitation volumes, {*s*_*i*_}. Black squares: surveyed results. Red circles: numerical results obtained at *γ*_*s*_. (**b2**) The variation of the normalized difference of city volume, Δ*s*, with respect to *γ*. Δ*s* is minimized at about ${\gamma }_{s}\approx 2.3$. (**c1**) The distribution of the tourist flows, {*w*_*ij*_} obtained from surveys. (**c2**) The variation of the normalized difference of tourist flows, Δ*w*, with respect to *γ*. Δ*w* is minimized at ${\gamma }_{L}\approx 3.5$. The parameter *γ* used in Eq. (1) is defined as the average of the three optimal parameters, $\gamma =({\gamma }_{L}+{\gamma }_{s}+{\gamma }_{w})\approx 2.9$.

The exponent *γ* in Eq. (1) is trained by comparing the results of numerical simulations and surveys for three statistical distributions: the route lengths ({*L*_*l*_}), the city visitation volumes ({*s*_*i*_}), and the tourist flows ({*w*_*ij*_}). The distribution of the route lengths as obtained from the surveyed results are plotted in Fig. 8(a1), where ${\rho }_{L}$ is the fraction of travel routes of length *L*. To find the optimal value *γ* that fits this distribution best, we scan *γ* over the range $(0,7)$ and, by numerical simulations, calculate for each value of *γ* the averaged difference between the the numerical and surveyed results, $\langle {\rm{\Delta }}\rho \rangle ={\sum }_{L=1}^{{L}_{max}}\,|{\rho }_{L}^{s}-{\rho }_{L}|/{L}_{max}$, with ${\rho }_{L}^{s}$ the fraction of routes of length *L* obtained by simulations and *L*_*max*_ the maximum travel length in the surveys. In Fig. 8(a2), we plot the variation of the normalized average difference, ${\rm{\Delta }}\rho =\langle {\rm{\Delta }}\rho \rangle /{\langle {\rm{\Delta }}\rho \rangle }_{max}$ (${\langle {\rm{\Delta }}\rho \rangle }_{max}$ is the largest difference in the scanned range), with respect to *γ*. We see that Δ$\rho$ reaches its minimum at ${\gamma }_{L}\approx 3.0$. We thus choose ${\gamma }_{L}=3.0$ as the optimal parameter trained from the distribution of route length.

The distribution of the city visitation volumes, {*s*_*i*_}, obtained from the surveys are plotted in Fig. 8(b1). To find the optimal parameter, *γ*_*s*_, for this distribution, we calculate the averaged difference between the surveyed and numerical results, $\langle {\rm{\Delta }}s\rangle ={\sum }_{i=1}^{n}\,|{s}_{i}^{s}-{s}_{i}|/n$, and plot in Fig. 8(b2) the variation of the normalized average difference, ${\rm{\Delta }}s=\langle {\rm{\Delta }}s\rangle /{\langle {\rm{\Delta }}s\rangle }_{max}$, with respect to *γ*. It is seen that Δ*s* is minimized at about 2.3. We therefore set ${\gamma }_{s}=2.3$. The distribution of the tourist flows, {*w*_*ij*_}, as obtained from the surveys are plotted in Fig. 8(c1). Defining the averaged difference as $\langle {\rm{\Delta }}w\rangle ={\sum }_{i > j}\,|{w}_{ij}^{s}-{w}_{ij}|/[{(n-1)}^{2}/2]$, we plot in Fig. 8(c2) the variation of the normalized average difference, ${\rm{\Delta }}w=\langle {\rm{\Delta }}w\rangle /{\langle {\rm{\Delta }}w\rangle }_{max}$, with respect to *γ* based on numerical simulations. We see that Δ*w* reaches its minimum at ${\gamma }_{w}\approx 3.5$. To balance between the three statistical distributions, we take their average, $\gamma =({\gamma }_{L}+{\gamma }_{s}+{\gamma }_{w})\approx 2.9$, as the final exponent used in Eq. (1).

## Statement

The questionnaire surveys were carried out in accordance with the guidelines and regulations of Shaanxi Normal University and approved by the Academic Committee of School of Geography and Tourism at Shaanxi Normal University. The informed consent was obtained from all participants and/or their legal guardians.

## Supplementary information

Supplementary

## Data Availability

The data that support the findings of this study are available from the corresponding author upon reasonable request.
