# Supplementary material for: Agent-based simulations of China inbound tourism network
Source: Sci Rep. 2019 Aug 23;9:12325. doi: 10.1038/s41598-019-48668-2 (PMC6707315; doi:10.1038/s41598-019-48668-2)
Supplement: Supplementary file 1 — Supplementary [file 41598_2019_48668_MOESM1_ESM.pdf]

Agent-based simulations of China inbound tourism network

Jinfeng Wu,[1] Xingang Wang,[2,*] and Bing Pan[3]

[1]*School of Geography and Tourism, Shaanxi Normal University, Xi'an 710062, China*

[2]*School of Physics and Information Technology,*

Shaanxi Normal University, Xi'an 710062, China

[3]*Department of Recreation, Park, and Tourism Management,*

School of Health and Human Development,

Pennsylvania State University, University Park, Pennsylvania 16802, USA

[*] E-mail: wangxg@snnu.edu.cn

I. SURVEY EXAMPLE

Figure S1 shows one of the questionnaires returned from the surveyed tourists. Key information in the questionnaire include: (1) the arrival and departure cities; (2) travel length; (3) days of stay; (4) travel planner; (5) travel purpose; (6) gender and nationality; (7) age; (8) income level; and (9) education level. The demographics of the final samples (856 in total) are given in Tab. S1.

II. SATURATION POINT OF TOURISM ATTRACTIVENESS

To saturation point is defined as the critical tourism attractiveness, a_c, of a city over which the increasing speed of the overall visitation volume with respect to the tourism attractiveness, $S'_{slope} = |\partial S/\partial a|$, is smaller to a small threshold, S'_c. To find a_c for a specific city, we increase a from its current value to 1×10^3, and calculate the variation of the overall visitation volume, S. The results for some major non-port cities are plotted in Fig. S2. We see that with the increase of a, the value of S is first increased with a fast speed, then is gradually saturated. Setting $S'_c = 0.1$, the saturation attractiveness of Xi'an [Fig. S2(a)], Guilin [Fig. S2(b)], Hangzhou [Fig. S2(c)], Chengdu [Fig. S2(d)], Suzhou [Fig. S2(e)], Kunming [Fig. S2(f)], and Chongqing [Fig. S2(g)] are, respectively, 755, 845, 670, 910, 500, 950, and 900.

TABLE S1. Demographics of the final samples.

Variable	Categories	Percent (%)
Gender	Male	39.53%
	Female	60.47%
Age	≤ 14	2.07%
	$15 - 24$	26.78%
	$25 - 44$	46.44%
	$45 - 64$	21.86%
	≥ 65	2.85%
Education	High school (or lower)	23.56%
	College	41.37%
	Master degree	28.51%
	Doctoral degree	6.56%
Income	High	17.39%
	Medium	34.72%
	Low	27.90%
Profession	Students	31.86%
	Technican	31.72%
	Retired	4.16%
	Business	18.70%
	Engineer	5.68%
	Homemaker	2.08%
	Official	4.02%
	Others	1.80%

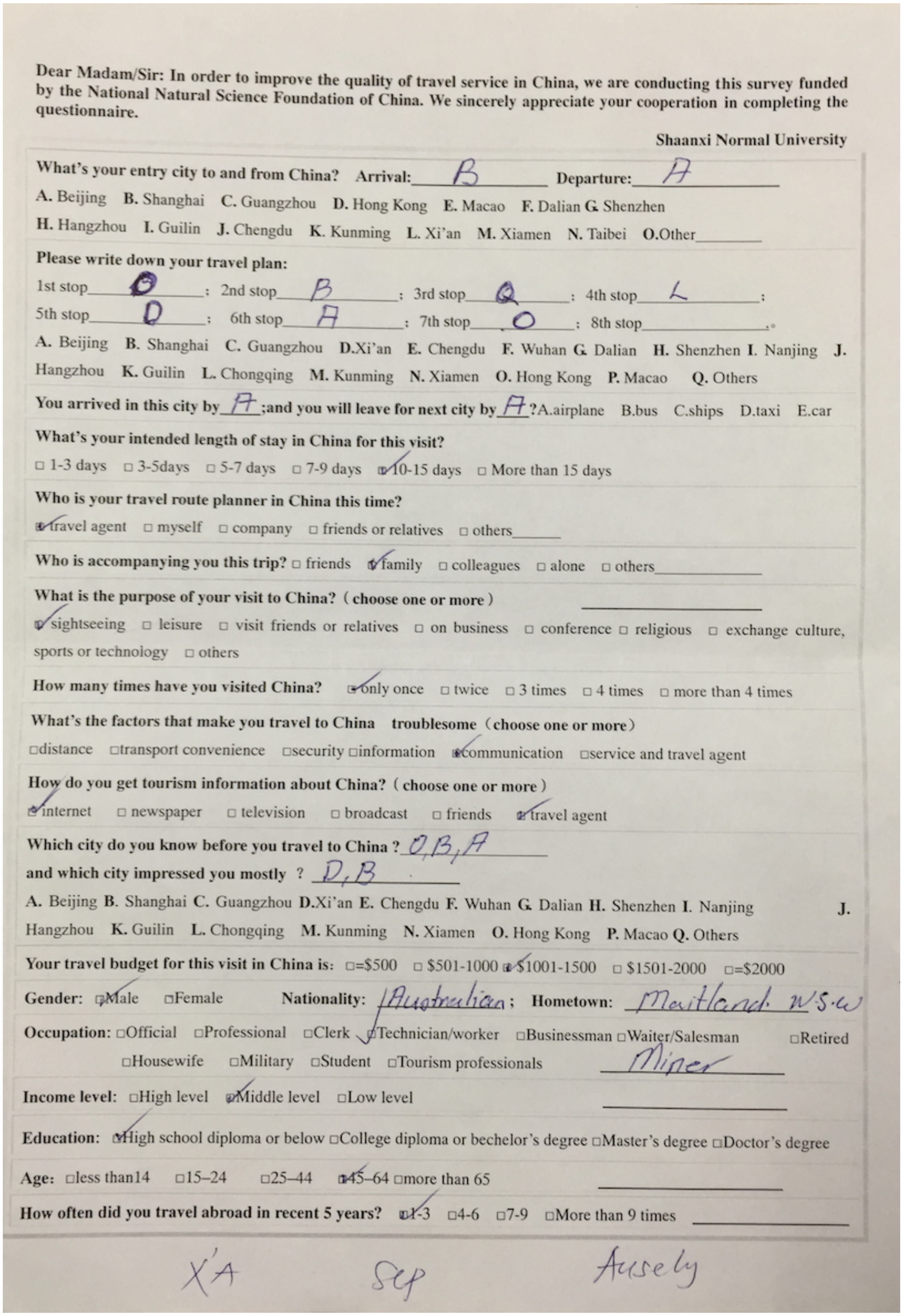

Dear Madam/Sir: In order to improve the quality of travel service in China, we are conducting this survey funded by the National Natural Science Foundation of China. We sincerely appreciate your cooperation in completing the questionnaire.

Shaanxi Normal University

What's your entry city to and from China? Arrival: B Departure: A

A. Beijing B. Shanghai C. Guangzhou D. Hong Kong E. Macao F. Dalian G. Shenzhen H. Hangzhou I. Guilin J. Chengdu K. Kunming L. Xi'an M. Xiamen N. Taibei O.Other____

Please write down your travel plan:

1st stop O; 2nd stop B; 3rd stop Q; 4th stop L; 5th stop D; 6th stop A; 7th stop O; 8th stop____。

A. Beijing B. Shanghai C. Guangzhou D.Xi'an E. Chengdu F. Wuhan G. Dalian H. Shenzhen I. Nanjing J. Hangzhou K. Guilin L. Chongqing M. Kunming N. Xiamen O. Hong Kong P. Macao Q. Others

You arrived in this city by A ;and you will leave for next city by A ?A.airplane B.bus C.ships D.taxi E.car

What's your intended length of stay in China for this visit?

□ 1-3 days □ 3-5days □ 5-7 days □ 7-9 days ☑ 10-15 days □ More than 15 days

Who is your travel route planner in China this time?

☑ travel agent □ myself □ company □ friends or relatives □ others____

Who is accompanying you this trip? □ friends ☑ family □ colleagues □ alone □ others____

What is the purpose of your visit to China? (choose one or more)

☑ sightseeing □ leisure □ visit friends or relatives □ on business □ conference □ religious □ exchange culture, sports or technology □ others

How many times have you visited China? ☑ only once □ twice □ 3 times □ 4 times □ more than 4 times

What's the factors that make you travel to China troublesome (choose one or more)

□distance □transport convenience □security □information ☑communication □service and travel agent

How do you get tourism information about China? (choose one or more)

☑ internet □ newspaper □ television □ broadcast □ friends ☑ travel agent

Which city do you know before you travel to China ? O,B,A

and which city impressed you mostly ? D,B

A. Beijing B. Shanghai C. Guangzhou D.Xi'an E. Chengdu F. Wuhan G. Dalian H. Shenzhen I. Nanjing J. Hangzhou K. Guilin L. Chongqing M. Kunming N. Xiamen O. Hong Kong P. Macao Q. Others

Your travel budget for this visit in China is: □=$500 □ $501-1000 ☑ $1001-1500 □ $1501-2000 □=$2000

Gender: ☑Male □Female Nationality: Australian; Hometown: Maitland N.S.W

Occupation: □Official □Professional □Clerk ☑Technician/worker □Businessman □Waiter/Salesman □Retired □Housewife □Military □Student □Tourism professionals Miner

Income level: □High level ☑Middle level □Low level

Education: ☑High school diploma or below □College diploma or bechelor's degree □Master's degree □Doctor's degree

Age: □less than14 □15–24 □25–44 ☑45–64 □more than 65

How often did you travel abroad in recent 5 years? ☑1-3 □4-6 □7-9 □More than 9 times

X'A Sep Ausely

FIG. S1. **An example of the returned questionnaires**.

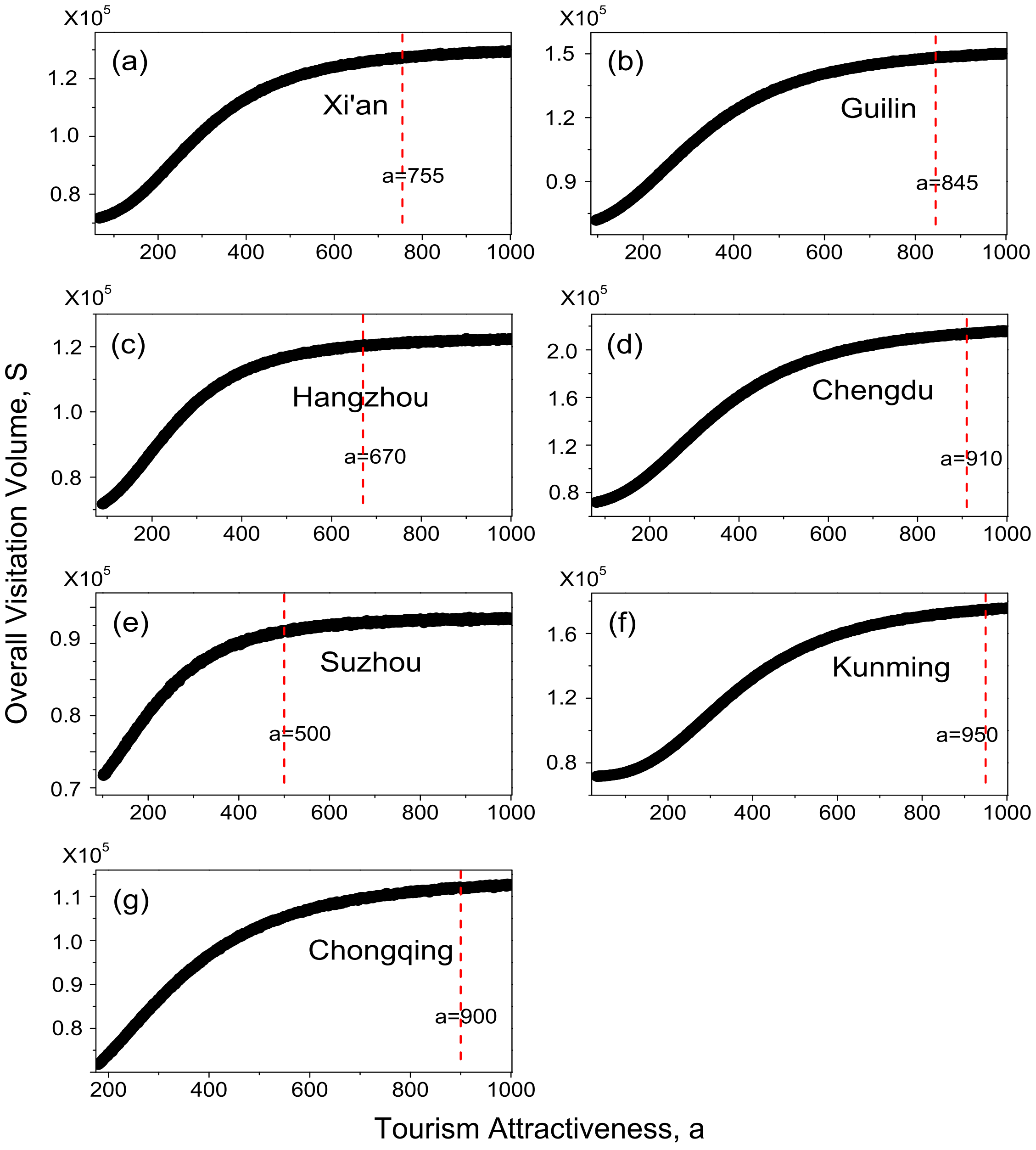

FIG. S2. **The saturation point of tourism attractiveness**. (a) Xi'an. $a_0 = 66$, $a_c \approx 755$. (b) Guilin. $a_0 = 96$, $a_c \approx 845$. (c) Hangzhou. $a_0 = 90$, $a_c \approx 670$. (d) Chengdu. $a_0 = 80$, $a_c \approx 910$. (e) Suzhou. $a_0 = 102$, $a_c \approx 500$. (f) Kunming. $a_0 = 32$, $a_c \approx 950$. (g) Chongqing. $a_0 = 180$, $a_c \approx 900$. The critical increasing speed is chosen as $S'_{slope} = 0.1$. a_0 denotes the current tourism attractiveness of the investigated city.
